# Spatio-Temporal Analysis of Micro Economic Activities in Rome Reveals Patterns of Mixed-Use Urban Evolution

**DOI:** 10.1371/journal.pone.0151681

**Published:** 2016-03-16

**Authors:** Alessandro Fiasconaro, Emanuele Strano, Vincenzo Nicosia, Sergio Porta, Vito Latora

**Affiliations:** 1 School of Mathematical Sciences, Queen Mary University of London, Mile End Road, E14NS London, United Kingdom; 2 Laboratory of Geographic Information Systems (LASIG), School of Architecture, Civil and Environmental Engineering (ENAC), Ecole Polytechnique Fédérale de Lausanne (EPFL), Lausanne, Switzerland; 3 Urban Design Studies Unit, University of Strathclyde, Glasgow, United Kingdom; 4 Dipartimento di Fisica ed Astronomia, Università di Catania and INFN, I-95123 Catania, Italy; National Scientific and Technical Research Council (CONICET)., ARGENTINA

## Abstract

Understanding urban growth is one with understanding how society evolves to satisfy the needs of its individuals in sharing a common space and adapting to the territory. We propose here a quantitative analysis of the historical development of a large urban area by investigating the spatial distribution and the age of commercial activities in the whole city of Rome. We find that the age of activities of various categories presents a very interesting double exponential trend, with a transition possibly related to the long-term economical effects determined by the oil crisis of the Seventies. The diversification of commercial categories, studied through various measures of entropy, shows, among other interesting features, a saturating behaviour with the density of activities. Moreover, different couples of commercial categories exhibit over the years a tendency to attract in space. Our results demonstrate that the spatio-temporal distribution of commercial activities can provide important insights on the urbanisation processes at work, revealing specific and non trivial socio-economical dynamics, as the presence of crisis periods and expansion trends, and contributing to the characterisation of the maturity of urban areas.

## Introduction

In present urbanism the idea that cities are mostly and essentially condenser of social and economic activities is popularly associated with Jane Jacobs’ call for more compact urban environments around socially inclusive open spaces [1]. It is today well known that cities emerge and grow because density pays off, and does that at a pace that largely off-sets that of its negatives [2–5]. The first step in understanding cities is therefore examining why activities are concentrated in a few places. Certain activities in fact exhibit an increasing return-to-scale, meaning that they profit proportionally more, or cost proportionally less, than the spatial growth of the city, and this is regarded as the driving force behind the growth of cities [6]. However, what makes cities great in expanding the benefit of concentration is not just the economy of scale per se, but that whatever you do occurs in a place where a number of other things also occur nearby at the same time. Those nearby activities may belong to the same industry (*localization economies*) or to a variety of different industries (*urbanization economies*). Understanding whether localization or urbanization economies are the reason why cities grow, and under what circumstances, is obviously very important to determine policies towards increasing specialization or rather, on the other hand, increasing heterogeneity.

Economic activities and the way they tend to locate in cities have been investigated from the point of view of spatial economics, looking at the equilibrium in space between factors like customers, suppliers and transport costs that would determine the optimal location of activities; this stream of studies, that draws back to classics written as early as in the late XIXth-early XXth century [7–9], has paved the way for an intense post-war period of progressive regional and urban analysis [10], which now struggles to make its way into a globalized world dominated by uncertainty, expanding urban poverty and the increasing fragmentation of decision making systems, including urban planning systems.

A more recent area of scientific investigation of urban economics has shifted the focus from business and employment only to retail, acknowledging the fundamental role of the sector in processes of urbanization and taking advantage of new potentials in Geographic Information Systems (GIS) modeling [11]. This stream of research refers directly to studies in marketing and business organization, largely a response to industry’s quest to develop scientific models of geo-located advantage with regards to potential market, closest competitors and other purely business management factors. Most importantly, this literature has grown on previous investigations of patterns of retail agglomeration that looked at retail diversification and at spatial features influencing rejection and attraction between categories of outlets [12–14]. In this context, the diversification of retail activities in an urban area is considered as an important aspect of the social “attractiveness” of a city, and it is becoming nowadays subject of detailed investigation.[15, 16].

Today, it is possible to study the location and diversity of commercial activities in large real-world urban systems, by making use of advanced GIS techniques and even mobile phone or geo-located online social networks [17–22]. Notwithstanding the recent progress in studying cities as spatial networks [23–27], a quantitative analysis of the historical development of the distribution of activities in a city is still missing, mainly because of the difficulties in getting historical data in electronic format. Building on a unique database, in this work we contribute a first empirical study of the development of retail activities in a city in time, by looking at the type and date of birth of all the activities present at year 2004 along with their geographical position. Specifically, the data set we have analysed contains the exact position in space (location), the year of registration and the market category (type) of each of the *N* = 35,053 commercial activities present in the city of Rome (Italy) at the year 2004. Notice that, since the first activity was registered on the 1^st^ of January 1900, while the last one on the 1^st^ of July 2004, the entire data set covers a period of more than a century. However, in our study we do not have complete information on the whole set of activities present in Rome at a certain time *t*, but only on those activities present at time *t* which survived up to year 2004. In short, of all activities that have populated the city of Rome in the analysis period, our data set only refers to the “survivors” at 2004, and we look backward to their behaviors in time as associated to their type and location. Each activity in fact belongs to one of eight commercial categories (or types). In Table 1 we report a list of the categories together with the number of activities *N*^*α*^ of each type *α*, with *α* = 1, 2, …, 8.

**Table 1:** Number *N*^*α*^ of commercial activities of type *α* in Rome at year 2004.

	Type *α*	Number *N*^*α*^
1	Not Specialised (not alimentary)	1946
2	Food	3898
3	Medicals	1640
4	Goods	16225
5	2^*nd*^ hand	130
6	Unconventional (mail retail, street selling)	4907
7	Repair	1166
8	Other	3025

## Results

### Double trend of temporal growth

We first looked at the temporal evolution of the number of activities which survived up to 2004. We considered both the total number of activities *N*(*t*) *present* at time *t* and still active at time 2004, and the number of new activities *n*(*t*) which were *registered* at time *t* and survived up to 2004. These two quantities are related through the expression $N\left(t\right)=\sum_{\tau =t_{0}}^{t}n\left(\tau \right)$ where *t*_0_ is equal to year 1900. More generally, in the following we will always use uppercase letters for quantities related to the total number of activities *N*(*t*) and lowercase letters for quantities related to the differential number of activities *n*(*t*).

**Fig 1:**
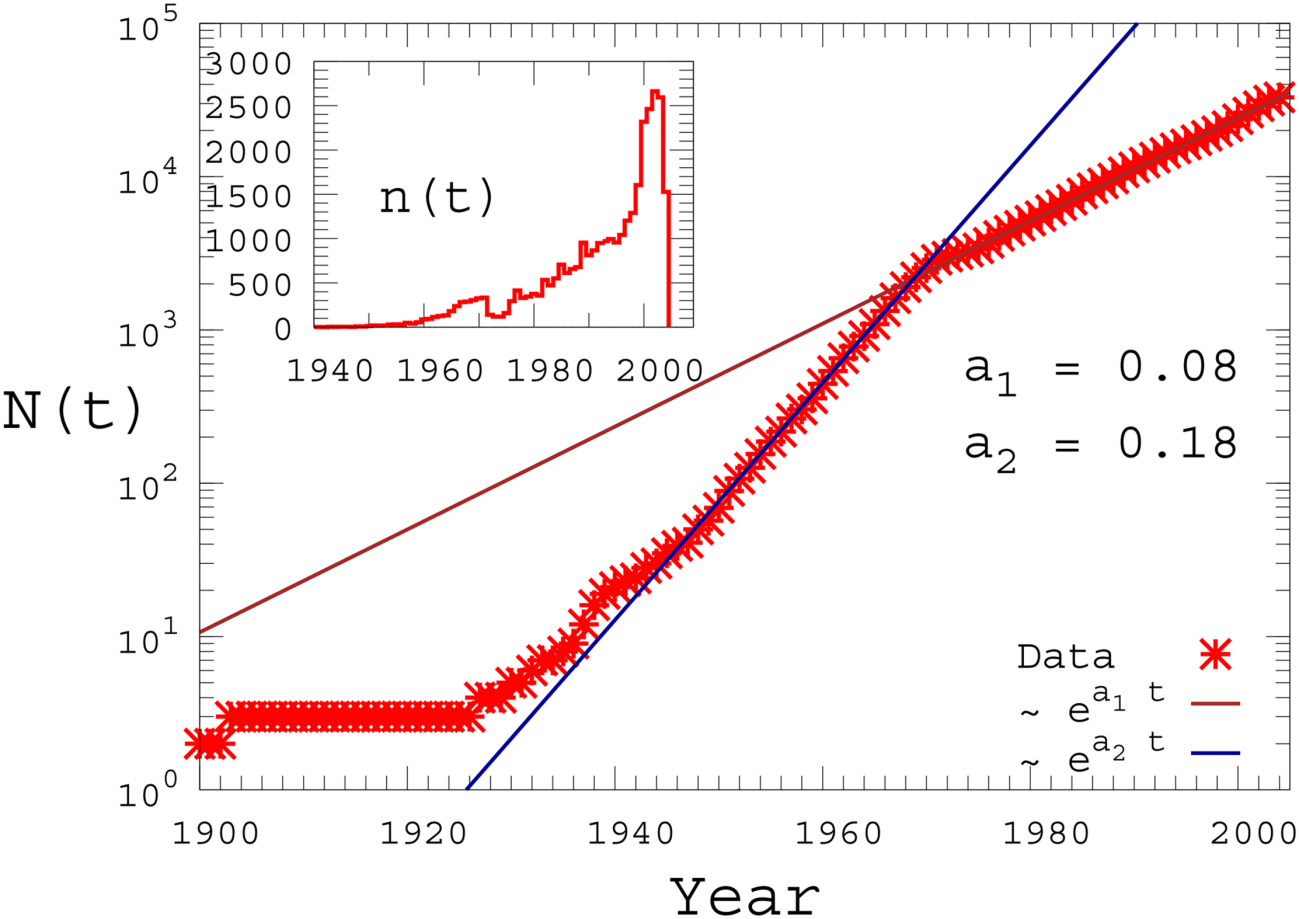
Temporal evolution of the number of commercial activities. Left: The cumulative number of survived activities, *N*(*t*), i.e. the number of activities present at time *t* in Rome, is plotted as a function of time *t* (expressed in calendar years). The inset shows the number *n*(*t*) of new registered activities at each year. Right: The number *n*^*α*^(*t*) of new activities is reported as a function of the year *t*, for a selection of the commercial categories reported in Table 1, i.e. Food, Medicals, Goods and Repair.

In the left panel of Fig 1 we report the number *N*(*t*) of activities present at time *t* as a function of the year *t* in a semi-logarithmic plot. We notice the presence of two well-defined exponential increases of the form *N*(*t*)∼*e*^*at*^, characterised by different values of the parameter *a*, respectively *a*_1_ = 0.08 and *a*_2_ = 0.18. The change of slope in *N*(*t*) occurs around the year 1975. In order to explain this double trend, we report in the inset the number *n*(*t*) of activities registered at each year *t* as a function of *t*, in a double-linear scale. We observe that the number of registered activities *n*(*t*) exhibits a sudden drop in the period 1973–1975. That time interval coincides with the first oil crisis, caused by the Yom Kippur Arab Israeli war and the consequent embargo declared by the Organisation of Arab Petroleum Exporting Countries (OAPEC) in October 1973, which had long-term effects not just on the price of petroleum but also, and more importantly, on all the major Western economies. The right four panels of Fig 1 report the number *n*^*α*^(*t*) of new activities registered each year for some relevant commercial categories, i.e. “Food”, “Medicals”, “Goods” and “Repair” (the behaviour of *n*(*t*) for the other categories is qualitatively similar). Notice that a drop in the number of new activities between 1973 and 1975 is visible, for instance, for “Food” and “Goods”, while other categories like “Medicals” and “Repair” do not show any sensible variation of the overall increasing trend. This first result is a tangible indication of how much the first oil crisis might have affected the increase of commercial activities in Western Europe. It appears that the crisis produced both a measurable decrease in the number of new activities, and a long-term change in the increase of the total number of activities after 1975, as testified by the sharp change of slope of *N*(*t*) in the left panel of Fig 1.

**Fig 2:**
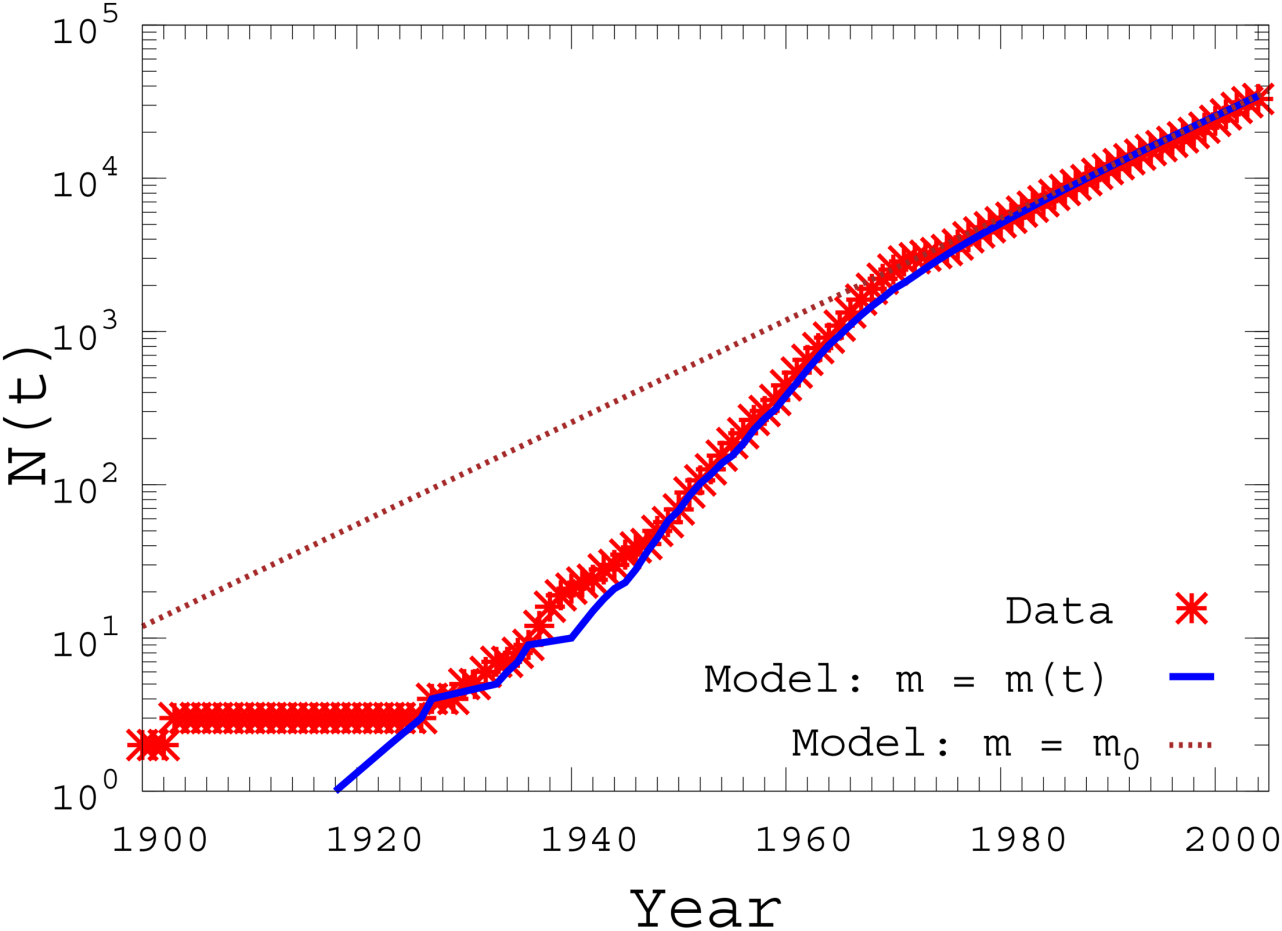
Modeling the double trend. The cumulative number *N*(*t*) of activities at time *t* that survived at year 2004 obtained by the model of birth-death described in the text is compared to the real data (symbols). The model produces an exponential distribution (dashed-line) when the number *m* of new activities added every year is constant, while gives rise to the empirically observed double trend when *m* is exponentially increasing in time until the year 1973 (full line).

In order to provide a quantitative explanation of the possible origins of the double trend behavior reported in Fig 1 we have considered the following model for the temporal evolution of the number of activities. The model is based on two mechanisms, the death of some of the existing commercial activities and the arrival of new ones. Namely, every year, starting from the year 1900, each of the existing activities dies with a probability *p*, while *m* new activities are added. This model can be solved analytically and produces an exponential distribution for the number of activities *N*(*t*) present at time *t*. The distribution obtained for *m* = 2500 and *p* = 0.077 is reported in Fig 2 as dashed line and correctly reproduces only the behavior observed in the city of Rome after the period 1973–1975. If we want to capture the double trend found empirically, we need to assume that the parameter *m* of the model changes over time. We have therefore assumed that *m* increases exponentially with the time, before the year 1973, while it stays constant in the period after the crisis. This is justified by the increasing prosperity of the city during the XIX century up to 1973. As for the growing rate parameter *α*_*m*_ in the function *m*(*t*)∝*e*^*α*_*m*_*t*^ we have used *α*_*m*_ = 0.11. The results of the numerical simulations of the model in this case are reported in Fig 2 as full lines. Notwithstanding its simplicity, the model takes into account of the long lasting impact generated by the crisis in the city of Rome, and results in very good agreement with the data.

### Diversification of activity types

The attractiveness of an urban area is quite often related to the diversity of resources made available to its inhabitants. When it comes to commercial activities, such variety implies the presence of retail shops belonging to several different categories. It is therefore interesting to explore how the relative number of activities of each category in our data set has evolved over time. We start by defining the probability $p^{\alpha }\left(t\right)=\frac{n^{\alpha }\left(t\right)}{n(t)}$ that a new activity of type *α*, *α* = 1, …, 8 is registered at time *t*, that is the ratio between the number of new activities of type *α* at *t* and the total number of activities registered at time *t*. Analogously, we define the probabilities $P^{\alpha }\left(t\right)=\frac{N^{\alpha }\left(t\right)}{N(t)}$, which refer to the total number of activities at time *t*.

**Fig 3:**
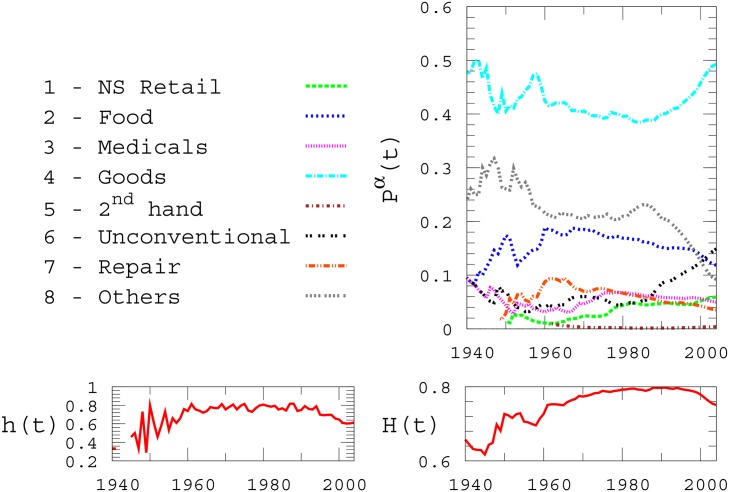
Time evolution of category diversification. Relative number of activities of type *α* present at time *t* (upper panel). Differential (left bottom panel) and cumulative (right bottom panel) entropies of category distributions are reported as a function of time *t*.

In the upper panel of Fig 3 we report the time evolution of *p*^*α*^(*t*) for each category *α*, starting at year 1940 (the points before *t* = 1940 were omitted from the plot due to the lack of sufficient statistics). Interestingly, even if the total number of activities keeps growing, as illustrated above, the relative abundance of categories remains approximatively constant during a long period of about 30 years, between 1960 and 1990. A reorganisation of the relative abundance of activities is instead observed in the period 1990–2004, and is mainly due to the drastic increase in the number of “Unconventional” activities and the concurrent decrease in the total number of activities in “Other”.

We used two measures to concisely quantify the heterogeneity of the activity distribution, namely the *category entropy* *h*(*t*), based on the registered activities at each time *t*, and the *cumulative category entropy* *H*(*t*). The first quantity is defined as:

$$h\left(t\right)=-C_{h}\sum_{\alpha =1}^{8}p^{\alpha }\left(t\right)\ln p^{\alpha }\left(t\right)$$ (1)

where *C*_*h*_ = 1/ln8 is a normalisation factor which guarantees that *h*(*t*) takes values in [0, 1]. The definition of *H*(*t*) is analogous, but is based on the cumulative distributions *P*^*α*^(*t*). The two entropies defined above can be used to characterise the overall variety of activities present in the city, ignoring the actual locations of retail shops (we will focus on spatial distributions of activities in the next Section). In particular, *h*(*t*) = 0 only if we have exactly one type of commercial activity, while we obtained *h*(*t*) = 1 when *p*^*α*^(*t*) is a uniform distribution in the space of categories, i.e. when there are exactly the same number of activities for each category.

The values of *h*(*t*) and *H*(*t*) are shown in the two bottom panels of Fig 3. After an initial phase characterised by a noisy increase of their values, both entropies reach a stable plateau which lasts for about three decades (roughly, between 1960 and 1990) and includes the overall maximum of each quantity over the whole data set. The presence of a plateau around a maximum value of entropy suggests that the attainment of a relatively well-balanced variety of activities after the WWII, corresponding with the economic growth in the 1950’s and 1960’s, has indeed evolved into a stable pattern that has lasted for a considerably long temporal interval, at least until the early 1990’s, in agreement with the stability of the relative abundance of activities observed in the top panel of the same Figure.

It is interesting to notice that both *h*(*t*) and *H*(*t*) have started decreasing in the mid-1990’s, revealing that the concentration of more recent activities is substantially different from that that observed between 1960 and 1990. Specifically, we can see that in the upper panel of Fig 2 both “Goods” and “Unconventional” register a big increase, while “Other” and “Food” register an evident decrease. More generally, we observe a first growth phase of the survived retails and their diversification just after the war (urban expansion), followed by a period of retail growth and diversity stability (urban maturity), and then finally by a last decade of much slower retail growth characterized by loss of diversity (urban stagnation) up to the year 2004 where the entropy values have not yet stabilised to a new equilibrium.

This decrease is possibly due to market distribution innovations such as the spread of large scale retail followed by emerging online styles of purchase. In addition to this, another possible explanation is related to the occurrence of another factor, namely the economical crisis of the Eighties in Italy, that reduced the purchasing power of the Italian working class, and, possibly, the variety of retail requests.

### The spatial organization of commercial activities

**Fig 4:**
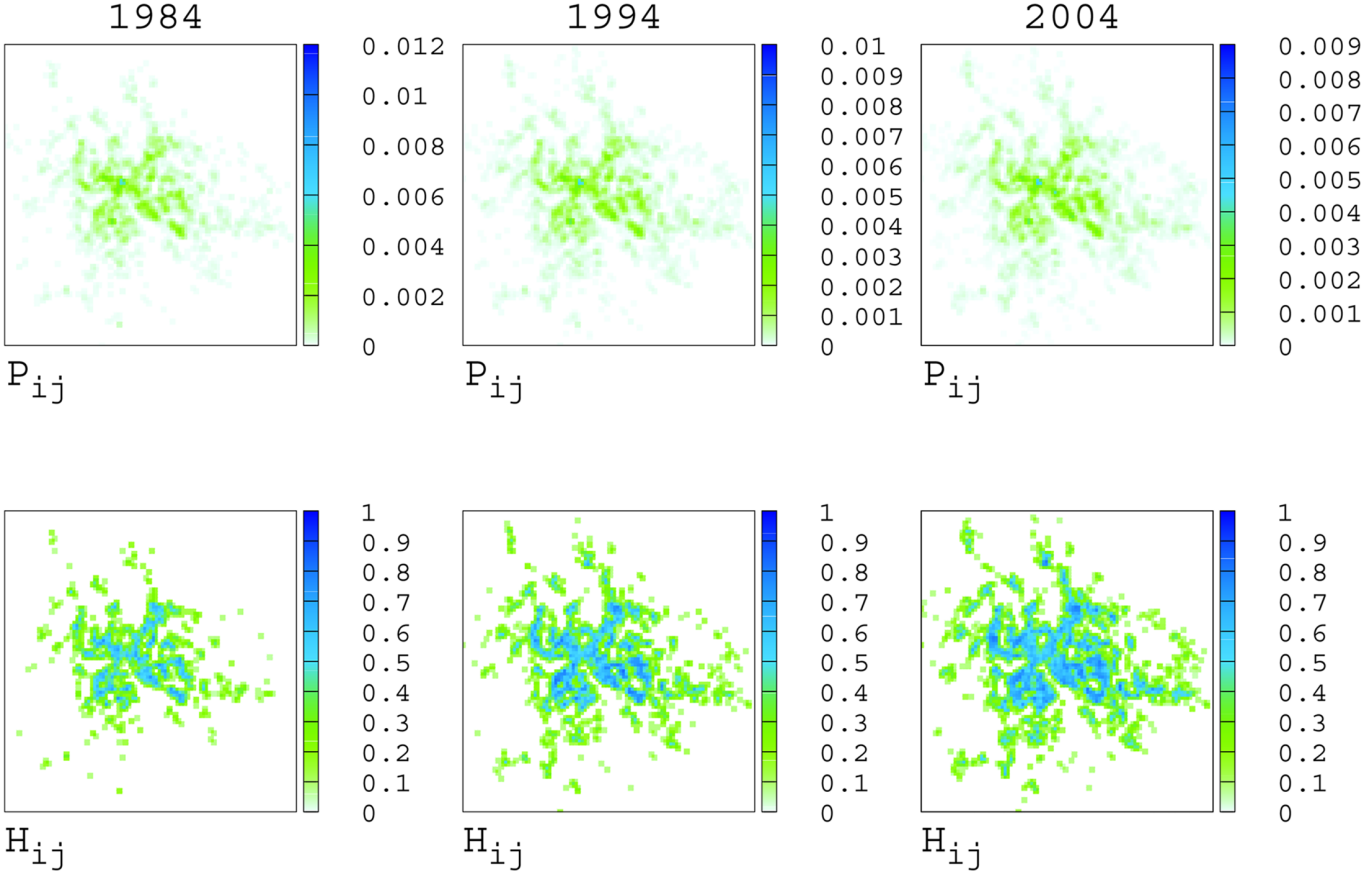
Spatial distribution of activities and category diversification. Spatial density of commercial activities (left/top panels), and distribution of the local category entropy *H*_*ij*_(*t*) (left/bottom panels) evaluated for the years 1984, 1994, and 2004 (1^st^, 2^nd^, and 3^rd^ column, respectively. We have considered a grid of *m* × *m* cells, with *m* = 100, corresponding to cells of linear size of 350 m. The two upper/right panels show the square root of the mean square displacement from the city centre of all the activities, respectively in the registered (*d*(*t*)) and cumulative (*D*(*t*)) cases. Panels on the right/bottom show the scatter plots of density, *P*_*ij*_(*t*), *vs* entropy, *H*_*ij*_(*t*) for the same three years.

An important element that characterises urbanization is how commercial activities and services are distributed in space. We considered a uniform grid of 100 × 100 square cells, each of length 350m, which covers all the activities present in the city council of Rome in 2004, and registered over the previous century. Each cell is identified by a pair of integer indices (*i*, *j*), with *i*, *j* = 1…100. We denote by *P*_*ij*_(*t*) the fraction of activities present at time 2004 which where present at time *t* inside the cell (*i*, *j*), and by *p*_*ij*_(*t*) the fraction of new activities registered at time *t* which fall within cell (*i*, *j*). In the upper-left panels of Fig 4 we show in a density plot the distribution *P*_*ij*_(*t*) at three points in time, namely in 1984, 1994, and 2004. It is clear that the spatial distribution of activities in the urban area has considerably evolved, with several zones becoming denser over time. Remarkably, the total area of the city in which we find retail activities has increased over time, as made evident by the plot of:

$$d\left(t\right)=\sqrt{\sum_{i}{\left[r_{i}\left(t\right)-r_{CM}\left(t\right)\right]}^{2}}$$

reported in the top-right panels of Fig 4. Notice that *d*(*t*) is the square root of the mean square displacement of the activity distributions at year *t* with respect to their centre of mass. The summation is extended over all the activities registered at time *t*, **r**_*i*_ ≡ (*x_i_*, *y_i_*) is the position of activity *i*, and $r_{CM}\left(t\right)=\sum_{i}r_{i}\left(t\right)$. We report also the temporal evolution of *D*(*t*), the analogous quantity computed considering the total number of activities present at time *t*.

We observe that, at the beginning of the past century, the activities present in Rome were confined within a circle whose radius was about 3 km. The behavior of *D*(*t*) is less affected by fluctuations and clearly shows the important geographical expansion of the city during the reconstruction period that followed the Second World War. Such an expansion lasted until the beginning of the 1970’s, and then gave rise to a period of 10–15 years when both *d*(*t*) and *D*(*t*) remained constant. A second relevant expansion of the city is visible in the decade 1985–1995, followed by a final stabilization lasting until 2004. The same trend is also observed when the quantity *D*(*t*) is computed for each of the different commercial categories (results not shown).

To better investigate the spatial distributions of the diversification of activities in Rome we have also considered the so-called *local category entropy*. If we denote by $p_{ij}^{\alpha }\left(t\right)$ the fraction of new activities within the cell (*i*, *j*) at time *t* which are of type *α*, the local category entropy of the cell (*i*, *j*) is defined as:

$$h_{ij}\left(t\right)=-C_{h}\sum_{\alpha =1}^{8}p_{ij}^{\alpha }\left(t\right)\ln p_{ij}^{\alpha }\left(t\right)$$ (2)

and quantifies how balanced is the distribution of categories in the cell. An analogous definition can be obtained from the cumulative distributions $P_{ij}^{\alpha }\left(t\right)$, and is denoted in the following as *H*_*ij*_(*t*).

The three panels in the bottom-left side of Fig 4 report as a colour-plot the values of *H*_*ij*_(*t*) for each cell, respectively in 1984 (left), 1994 (middle), and 2004 (right). In general, we observe a form of urbanization that proceeds establishing local centres with higher diversity of retail around which places with lower diversity contextually emerge as a “grey” background area. That seems to happen across scales, in a modular fashion which is reminiscent of long established theories of urban morphology [28].

The three panels on the bottom/right of Fig 4 report the scatter plots of *P*_*ij*_(*t*) *vs* *H*_*ij*_(*t*), again for the years 1984, 1994, and 2004. These plots exemplify the relationship between accumulation and diversification in the city, since *P*_*ij*_(*t*) and *H*_*ij*_(*t*) are measuring, respectively, the concentration of commercial activities in a cell and the heterogeneity of the categories in the same cell. Albeit the two measures are somehow correlated, it is interesting to notice that, for low values of *P*_*ij*_, the corresponding values of *H*_*ij*_ increase almost linearly while, for higher densities, the local category entropy saturates, supporting the idea that a high diversification of categories requires a critical density of activities to be achieved. The higher is the number of cells with large values of entropy *H*_*ij*_, the wider are the opportunities locally available to city users.

**Fig 5:**
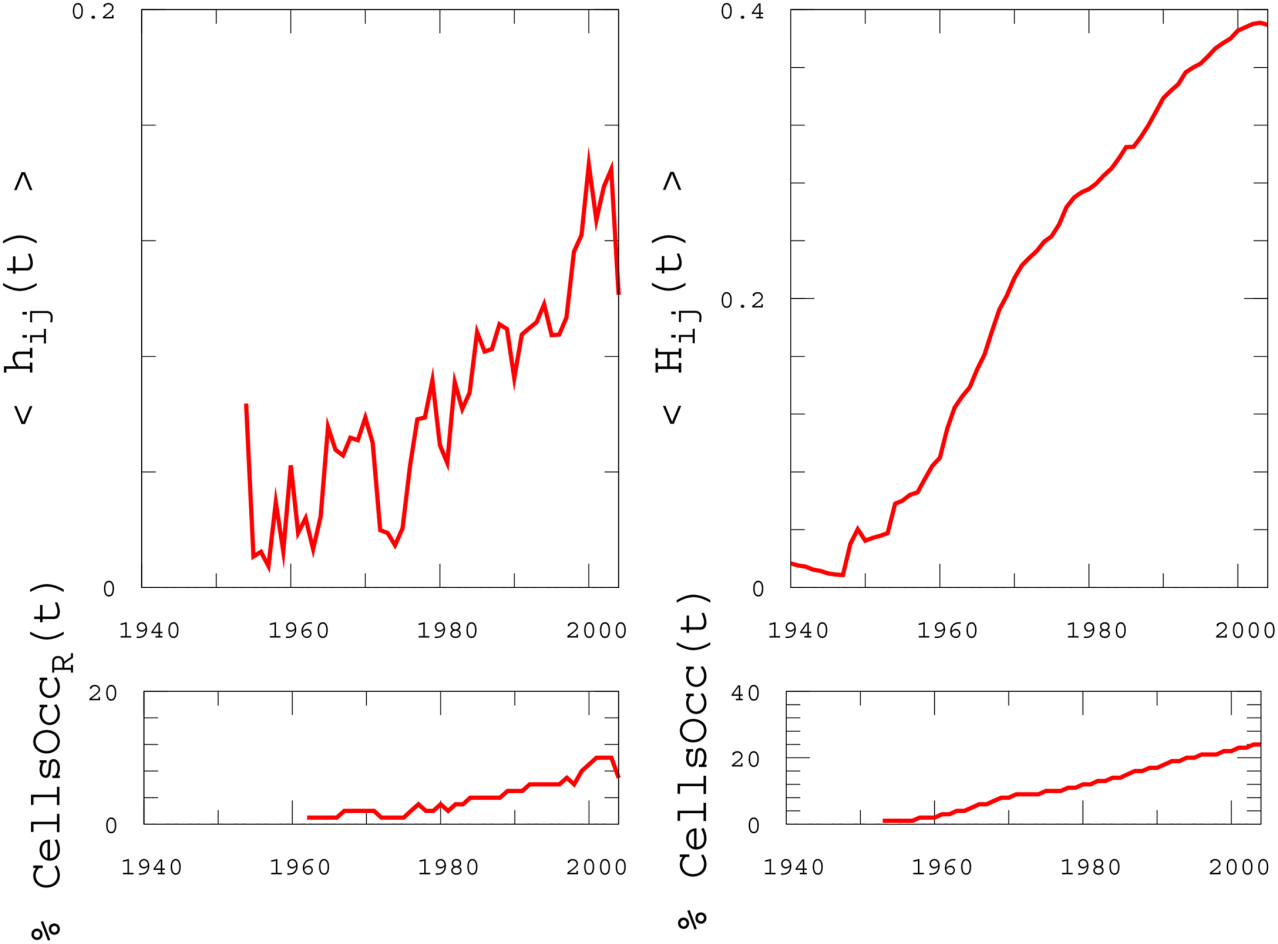
Local properties. The local activity entropy averaged over all the cells of the spatial grid (upper panels) in its differential (left) and cumulative version (right). Lower panels: Percentage of number of occupied cells of the grid.

A more compact information can be extracted from Fig 5, whose upper panels show the average values 〈*h*_*ij*_(*t*)〉 and 〈*H*_*ij*_(*t*)〉 of local category entropies, where the averages are computed over all the cells of the grid. Interestingly, both these entropies do not show the saturation observed in Fig 3 for *h*(*t*) and *H*(*t*), but are instead characterised by an overall increasing trend over the whole period of interest, revealing that different levels of diversification took place at both global and local scales. Notice that the effects of the first oil crisis are again visible in the plot of *h*_*ij*_(*t*) as a pronounced drop between 1973 and 1975, suggesting that the crisis affected the distribution of retail activities at different spatial scales. Finally, the lower panels of Fig 5 show the percentage of the cells occupied by, respectively, at least one new registered activity (left) and at least one activity (right). Even in this elementary measure, the Oil Crisis period results evident as a slight gap on the left plot and a small plateau on the right one.

### The network of attraction-repulsion between categories

Important information on the spatial distribution of activities in a city can be extracted by looking at how different commercial categories attract or repel each others, and how such attraction/repulsions patterns have evolved over the years. As proposed by Pablo Jensen in Ref. [14], a nice way to capture the correlations between the spatial position of commercial activities in a city is to construct a network where each node represents a different commercial category, and where the links stand for attraction/repulsion in space between couple of categories. We have constructed such networks for each year *t*, starting from the spatial distribution of activities, in the following way. Given two activity types, namely *α* and *β*, for each activity *i* of type *α*, we consider a circle of radius *R* centered at the position **r**_*i*_ = (*x_i_*, *y_i_*) of *i*. Being $N_{i}^{\beta }\left(t\right)$ and *N*_*i*_(*t*) respectively the number of activities of type *β* and the total number of activities inside the circle centered in **r**_*i*_ at time *t*, we consider the ratio $N_{i}^{\beta }\left(t\right)/N_{i}\left(t\right)$. We then compare $N_{i}^{\beta }\left(t\right)/N_{i}\left(t\right)$ to the expected value of the ratio, *N*^*β*^(*t*)/*N*(*t*), that we would obtain if the commercial activities of type *β* were distributed uniformly in space, independently from the positions of the activities of type *α*. If $N_{i}^{\beta }\left(t\right)/N_{i}\left(t\right)$ is different from *N*^*β*^(*t*)/*N*(*t*), this means that the presence of the activity *i* of type *α* at position **r**_*i*_ affects the presence of activities of type *β* in its vicinity. We hence define the *attraction coefficient* $A^{\alpha \beta }\left(t\right)$ between type *α* and type *β* at time *t* as the logarithm of the ratio between $N_{i}^{\beta }\left(t\right)/N_{i}\left(t\right)$ and *N*^*β*^(*t*)/*N*(*t*), averaged over all circles centered around the *N*^*α*^(*t*) activities of type *α*. We finally get the following expression [14]:

$$A^{\alpha \beta }\left(t\right)=\ln\left[\frac{1}{N^{\alpha }\left(t\right)}\frac{N(t)}{N^{\beta }\left(t\right)}\sum_{i=1}^{N^{\alpha }\left(t\right)}\frac{N_{i}^{\beta }\left(t\right)}{N_{i}\left(t\right)}\right]$$ (3)

**Fig 6:**
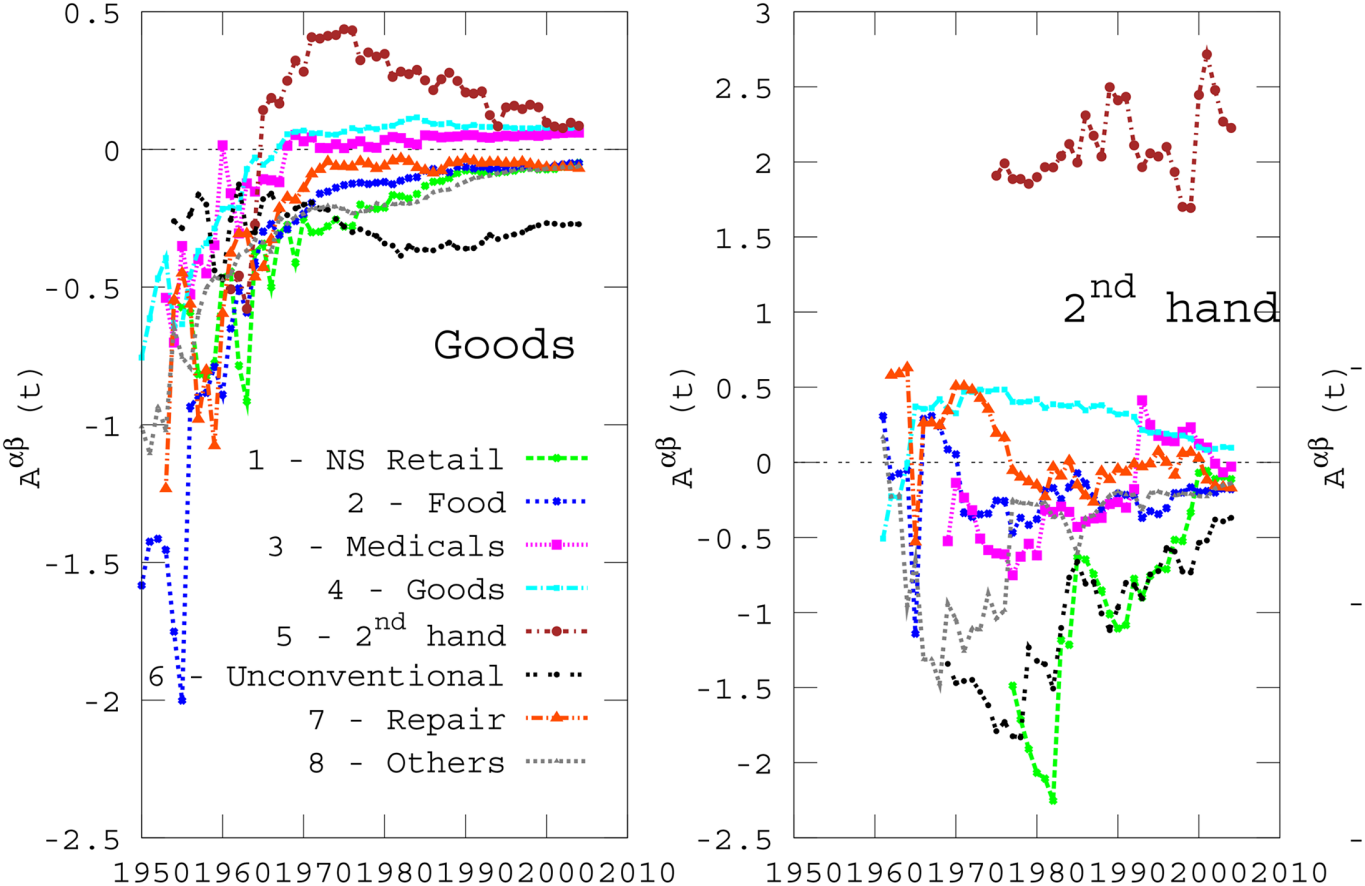
Time evolution of attraction between categories. The three panels show the values of the attraction coefficients $A^{\alpha \beta }\left(t\right)$ respectively for *α* = 4 (“Goods”), *α* = 5 (“2^*nd*^ hand”), and *α* = 7 (“Repair”), with respect to all the other categories, namely *β* = 1, …, 8. The radius *R* was set equal to 200 meters.

**Fig 7:**
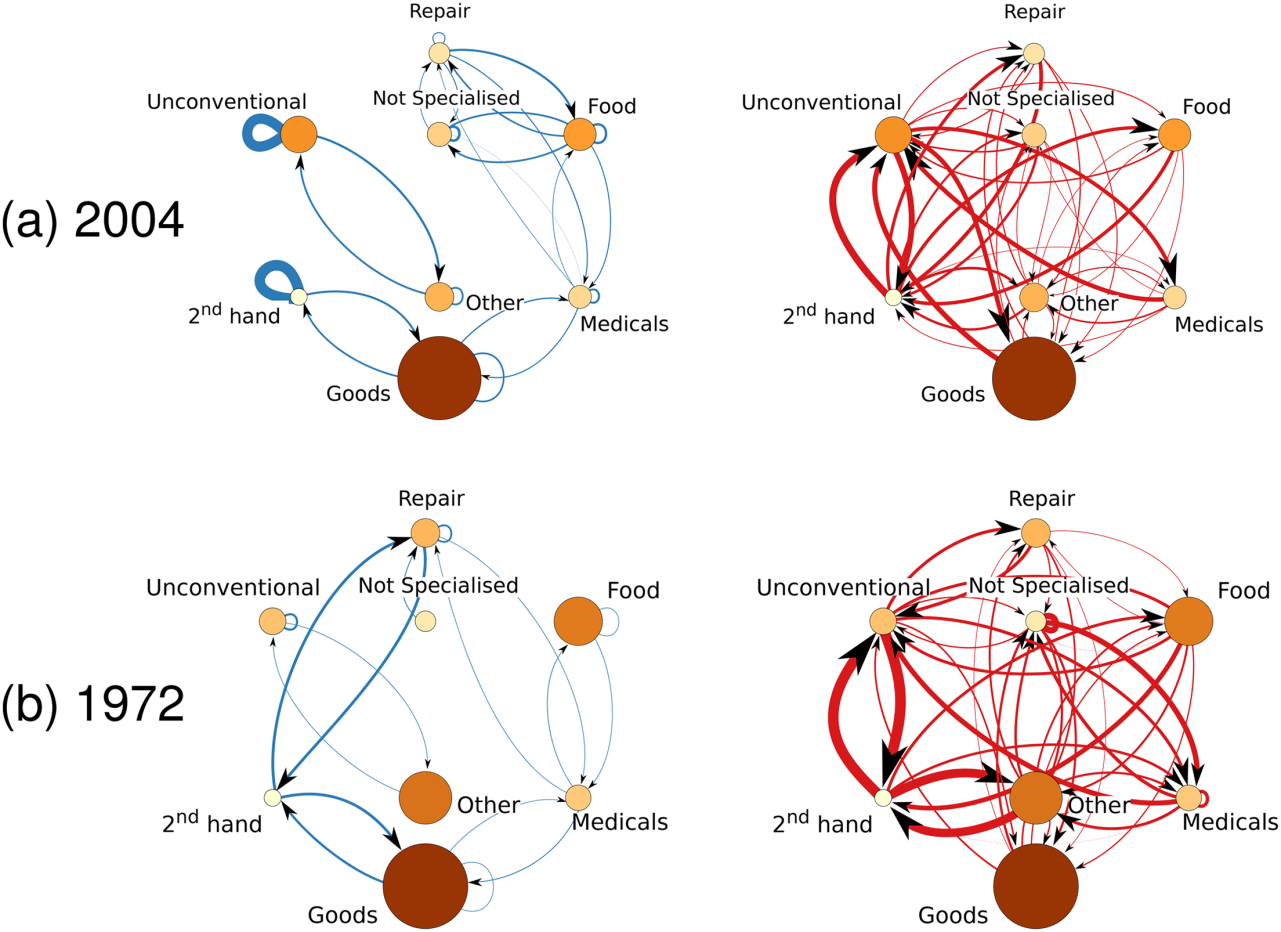
Networks of attraction and repulsion among activity types. The networks obtained from the spatial distribution of activities at year 2004 (panel (a)), and at year 1972 (panel (b)). The width of link *α* → *β* from category *α* to category *β* represents the value of $|A^{\alpha ,\beta }|$. Blue links indicate attractions (networks on the left hand side), while red links indicate repulsions (networks on the right hand side). The dimension of the nodes is proportional to the fraction of commercial activities in the corresponding category.

In this way, positive values of the attraction coefficient $A^{\alpha \beta }\left(t\right)$ indicate that the local density of activities *β* inside circles of radius *R* centered at activities of type *α* is higher than the average, and so category *α* attracts *β*. Conversely, negative values of $A^{\alpha \beta }\left(t\right)$ mean that *α* repels *β*, being the density of *β* inside circles centered at *α* lower than the average. In the three panels of Fig 6 we plot the attraction coefficients of three categories, namely “Goods”, “2^*nd*^ hand”, and “Repair”, with respect to each of the other eight categories as a function of time, where we considered *R* = 200 meters. The global pattern of attraction/repulsion at a given year can be represented and visualised in a graph. In Fig 7(a) we report the attraction (left) and repulsion (right) graph corresponding to year 2004. Each node represents a commercial category, and its size is proportional to the fraction of activities belonging to the corresponding category. The directed link from node *α* to node *β* indicates attraction (solid blue lines) or repulsion (dashed red lines) between the corresponding categories, and the width of an edge is proportional to the absolute value of the attraction coefficient $A^{\alpha \beta }$.

We found that in most of the case, except for the few self-loops, attraction edges are relatively weak, and in small number, while repulsion is usually much stronger, for instance for the pairs “2^*nd*^ hand-Unconventional”, “Goods-Unconventional”, and “Medicals-Unconventional”. Interestingly, several categories are characterised by self-attraction, as demonstrated by the presence of many blue loops, and can be extremely strong, such as in the case of “Unconventional” and “2^*nd*^ hand”. Conversely, there is no big repulsion loops. As a consequence, it seems that in general commercial activities of a given type tend to attract other activities of the same type. Although the attraction and repulsion graphs are directed, we remark that in most of the cases edges are reciprocated, meaning that if a link exists from a node *α* to a node *β*, then there is also a link from *β* to *α*, and the two links have a comparable weight. It is interesting to notice that generally we do not observe the concurrent presence of an attraction edge from *α* to *β* and a repulsion edge from *β* to *α*, although such configurations are possible in theory.

As a comparison, we report in Fig 7(b) the network of attraction/repulsion at year 1972, namely before the oil crisis. We notice that some of the relationships have been maintained over the years, possibly with small changes in their weight, while some are very different from the 2004 network. One example of relevant persisting relationship is that of the pair “Unconventional-2^*nd*^ hand”, which was repulsive in 1972 and remained repulsive in 2004, though with slightly different intensities. In other cases some links are, instead, not present at one year, but appear in the other; examples are the couples “Not Specialised-Repair”, or “Food-Medicals” which attract at the year 2004, but have a slight asymmetry in the behavior in 1972, where “Not Specialised” attracts “Repair”, but, curiously, “Repair” repulse “Not Specialised”.

It is interesting to notice that some attractive relationships might turn into repulsive ones over time and vice-versa. A typical example is the relation between “Repair-2^*nd*^ hand”. In fact, we can see from the third panel of Fig 6 that “Repair” attracts “2^*nd*^ hand” before the year 1970, while the opposite occurred after 1970. This behavior is also well visible in the network representation of Fig 7, where we can see in panel (b) left of year 1972 a very clear blue line between the two categories, while in the year 2004 the links between the couple are red (panel (a) right). A similar situation occurs for the couple “Not Specialised-Food” with a change from weak repulsive to attractive around the same year.

The same is also valid for some self-attractions. An example is given by the category “Goods”, which was self-repulsive before the 1970s, becomes self-attractive after 1970 (see the first panel of Fig 6), and the category “Not Specialised”, which flipped from a clear self-repulsion into a weak self-attraction around 1980. Table 2 reports the most important changes in the sign of pairwise relationships observed in the data set. It is there evident that most of the changes take place in one of three main periods, namely between the end of the 1960s and the early 1970s, around the middle of the 1980s, and around year 1993, when the changes have been weak but quite persistent in subsequent times. Interestingly, most changes are from repulsion to attraction. The opposite direction occurs only for the commercial type “Repair”, which was self-attractive before the year 1988, and self-repulsive afterwards for a couple of years, and very slightly self-attractive again in the last years before 2004.

**Table 2:** Summary of the most relevant changes in the sign of attraction between categories. The changes are generally symmetrical with respect to the two categories.

*α*–*β*		≈ year	Change
1–2	Not Specialised—Food	1970	− → +
2–1	Food—Not Specialised	1970	− → +
3–4	Medicals—Goods	1967	− → +
4–3	Goods—Medicals	1967	− → +
4–4	Goods—Goods	1966	− → +
8–8	Other—Other	1986	− → +
1–1	Not Specialised—Not Specialised	1983	− → +
7–7	Repair—Repair	1988	+ → −
1–7	Not Specialised—Repair	1993	− → +
3–3	Medicals—Medicals	1993	− → +
3–5	Medicals—2^*nd*^ hand	1993	− → +
5–3	2^*nd*^ hand—Medicals	1993	− → +
7–1	Repair—Not Specialised	1993	− → +

It is important to notice that the attraction coefficient we have considered compares the local concentration of activities with respect to the concentration in the whole city. Due to this normalisation, the evaluation of the attractiveness at year *t*, based on the spatial distribution at *t* of only those activities which survived up to year 2004, can give information of the properties of all the activities at year *t* (under the assumption that non-surviving activities have spatial distributions similar to those of the surviving activities).

Fig 6 also shows a slightly tendency towards the reduction of the value of |A| in time, and its stabilization to a relatively low value. In fact, the commercial activities start to become more evenly distributed in the territory, and only a weak coexistence or repulsion of different categories can be registered. This behaviour cannot be attributed to the increase of the number of activities, because, as visible in Fig 3, the density of activities is approximately constant in time and the small variations observed in the density do not affect the values of attractiveness.

## Discussion

In this paper we investigate location and type of all the 35,000 retail activities present in the city of Rome in 2004, along with their development in time over approximately one century, from 1900 to 2004. The aim of the study is to shed some light on the temporal dynamics of retail as one of the most fundamental drivers of urbanization.

In order to do so, we first look at the number of activities both in terms of registrations and cumulative presence year-by-year. We notice that while activities restlessly grow over the whole period, they do so at two very different speeds, with a marked change in speed emerging in the middle of the Seventies. This change immediately follows the first oil crisis, with “Repair” and “Medicals” being the only two types of activities that do not show any drop in registrations during the crisis. This tells the story of an abrupt shift in the conditions of the local market from a state of expansion to one of stagnation subsequent to global political moves.

We then look at the diversity of retail types first globally, i.e. the overall contribution of each type to the whole retail stock present in 2004 year by year over the period, and locally, i.e. their contribution year by year to the stock located in each square cell of 350 meters of edge of a virtual grid covering the whole city of Rome. We find that while the number of activities grows with time, their diversity manifests a threefold global behaviour: it grows in the years of the post-war reconstruction and expansion, remains quite stable after the Sixties for about three decades, and decreases in the last decade. Locally, however, we see a different scenario, where on one hand the higher diversity of retail activities occurs where their spatial concentration is higher, while on the other there does not appear to be any visible plafond to the local growth of retail diversity. The observed difference in the global and local organisation of activities, with the diversity of activity types continuing steadily to grow locally in a condition of global stability, can be of great importance in understanding the capacity of cities to find smaller scale forms of organisation that would normally go unnoticed.
