# Correction: Spatio-Temporal Analysis of Micro Economic Activities in Rome Reveals Patterns of Mixed-Use Urban Evolution

**DOI:** 10.1371/journal.pone.0154180

**Published:** 2016-05-11

**Authors:** The PLOS ONE Staff

The images for Figs 1, 4, and 6 are erroneously truncated. The publisher apologizes for the errors. Please see the corrected Figs 1, 4, and 6 here.

**Fig 1:**
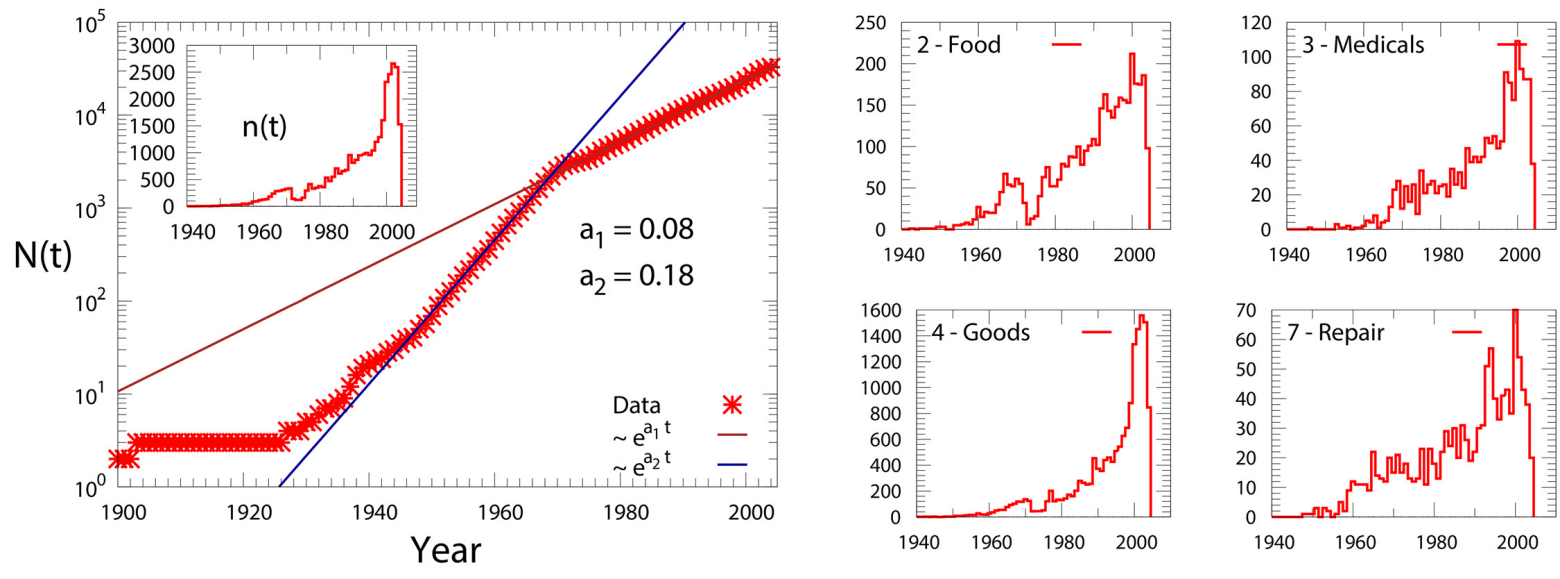
Temporal evolution of the number of commercial activities. Left: The cumulative number of survived activities, *N(t)*, i.e. the number of activities present at time *t* in Rome, is plotted as a function of time *t* (expressed in calendar years). The inset shows the number *n(t)* of new registered activities at each year. Right: The number *n*^*α*^*(t)* of new activities is reported as a function of the year *t*, for a selection of the commercial categories reported in Table 1, i.e. Food, Medicals, Goods and Repair.

**Fig 4:**
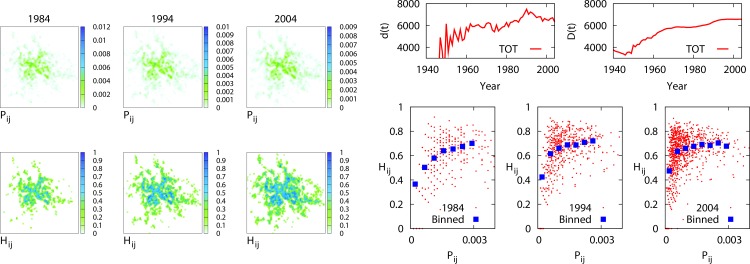
Spatial distribution of activities and category diversification. Spatial density of commercial activities (left/top panels), and distribution of the local category entropy *H*_*ij*_*(t)* (left/bottom panels) evaluated for the years 1984, 1994, and 2004 (1^st^, 2^nd^, and 3^rd^ column, respectively. We have considered a grid of *m × m* cells, with *m* = 100, corresponding to cells of linear size of 350 m. The two upper/right panels show the square root of the mean square displacement from the city centre of all the activities, respectively in the registered *(d(t))* and cumulative *(D(t))* cases. Panels on the right/bottom show the scatter plots of density, *P*_*ij*_*(t)*, vs entropy, *H*_*ij*_*(t)* for the same three years.

**Fig 6:**
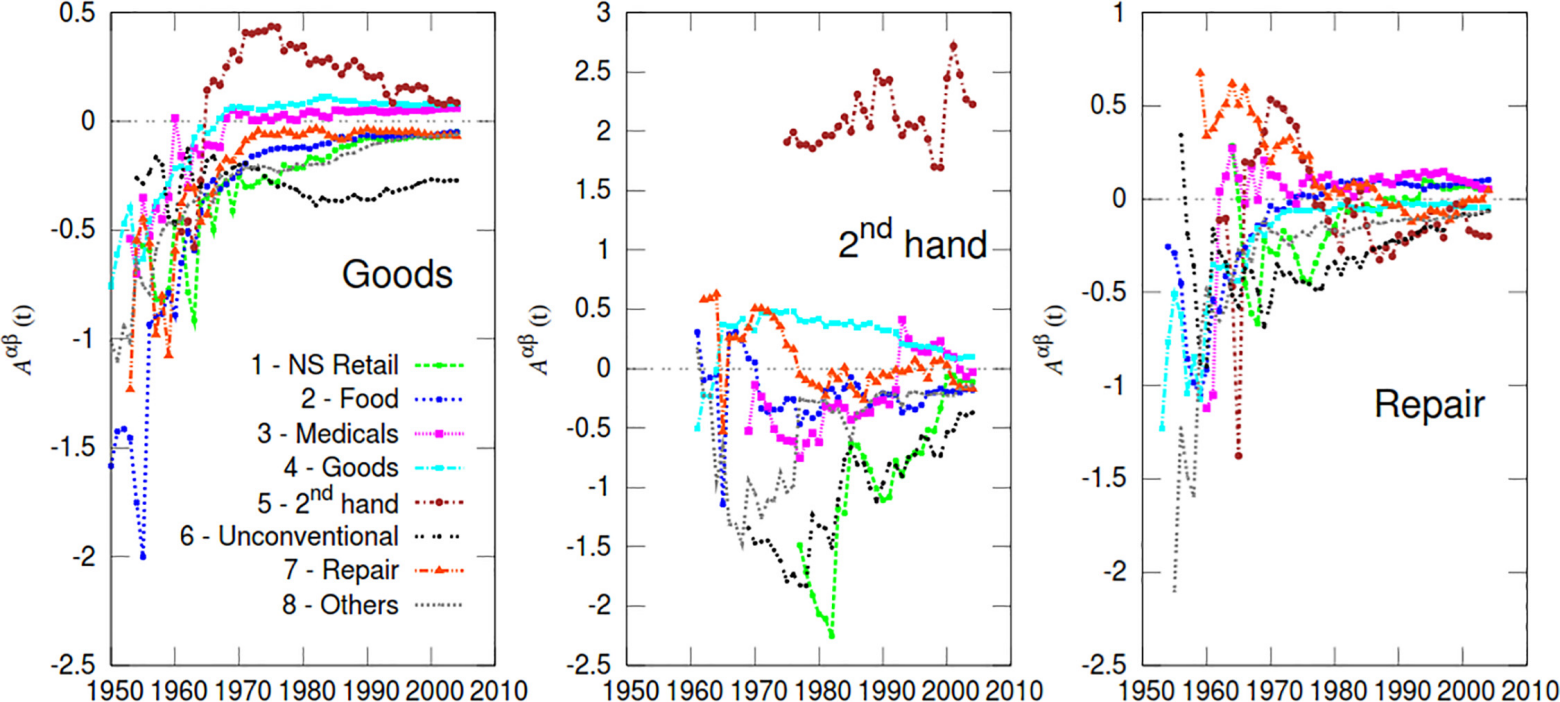
Time evolution of attraction between categories. The three panels show the values of the attraction coefficients $A^{\alpha \beta }\left(t\right)$ respectively for α = 4 (“Goods”), α = 5 (“2^*nd*^ hand”), and α = 7 (“Repair”), with respect to all the other categories, namely *β* = 1, …, 8. The radius *R* was set equal to 200 meters.
